# Impact of Neoadjuvant Approach and Tumor Response on Survival in Pancreatic Adenocarcinoma

**DOI:** 10.1002/jso.70329

**Published:** 2026-09-03

**Authors:** Mohammed O. Suraju, Brian Longbottom, Jasmine A. Peterson, Terry Hayes, Kiers Brevik, Sean Y. Kim, Paolo Goffredo, Imran Hassan, Hassan Aziz, Carlos H. F. Chan

**Affiliations:** ^1^ Department of Surgery University of Iowa Hospitals and Clinics Iowa USA; ^2^ Carver College of Medicine University of Iowa Iowa USA; ^3^ Department of Biology University of Iowa Iowa USA; ^4^ Department of Surgery University of Minnesota Minnesota USA; ^5^ Mercy Hospital Cedar Rapids Iowa USA

**Keywords:** multi‐agent, neoadjuvant, pancreatic adenocarcinoma, pathologic complete response, single‐agent, survival

## Abstract

**Introduction:**

Neoadjuvant therapy for pancreatic adenocarcinoma (PDAC) has evolved from single‐agent to multi‐agent approaches, largely informed by metastatic disease studies demonstrating improved survival; albeit at the price of increased toxicity. It remains unclear how response to varied neoadjuvant regimens impact survival. We hypothesized that tumor response to different neoadjuvant approaches reflects underlying tumor biology, and achieving a pathologic complete response (pCR) with single‐agent neoadjuvant may signal favorable biology.

**Methods:**

Adult PDAC patients (2010–2021) who received neoadjuvant therapy were identified in the National Cancer Database. Tumor response was categorized as pCR (pT0N0), moderate (≥ 2 T‐stage reduction), or minimal/no response (≤ 1 T‐stage reduction). Patients with stage IV disease, margin positive, or nodal involvement on final pathology were excluded; sensitivity analyses including these patients were performed. Neoadjuvant regimens were classified as single‐agent, multi‐agent, or multi‐agent therapy plus adjuvant chemotherapy.

**Results:**

Of 1923 patients, 9.8% achieved pCR, 19.1% had moderate response and 71.1% had minimal/no response. The 3‐year overall survival (OS) was 74% [68–81] for pCR, 56% [51–62] for moderate, and 50% [47–52] for minimal/no response. Notably, 11% of pCR patients received single‐agent therapy and had a 3‐year OS comparable to those who received more intensive regimens, even after adjusting for confounders. Conversely, single‐agent therapy in moderate or minimal/no responders was associated with significantly worse survival. Sensitivity analyses including patients with nodal metastasis and positive margins demonstrated consistent trends.

**Conclusions:**

Most PDAC patients benefit from multi‐agent neoadjuvant, although a small subset achieve pCR with single‐agent therapy and experience excellent outcomes. Response to varied neoadjuvant approaches merits further investigation in biomarker‐driven studies and may inform opportunities for therapy personalization in appropriately selected PDAC patients.

## 1 Introduction

Pancreatic ductal adenocarcinoma (PDAC) remains an aggressive disease with a 10‐year overall survival (OS) of less than 4% [1]. In fact, it is projected to be the second leading cause of cancer‐related deaths by 2030 [2]. Even among patients who undergo resection with curative intent, most die of metastatic disease rather than local recurrence within 2 years suggesting that PDAC is a systemic disease [3, 4].

Given the success of neoadjuvant therapy in several cancers such as esophageal, gastric, and rectal cancer, neoadjuvant approach is increasingly being used in PDAC [5, 6, 7]. Between 2014 and 2020, the proportion of patients receiving neoadjuvant therapy increased from about 24% to 47% in the US [5, 7]. Potential advantages of neoadjuvant approach include early eradication of micrometastatic disease, improved completion rates of systemic therapy, higher R0 resection rates, and better patient selection for surgery [8].

Nevertheless, the optimal neoadjuvant regimen remains controversial. Prior to 2010, most patients receiving neoadjuvant received single‐agent neoadjuvant, typically gemcitabine. However, the multi‐agent regimen FOLFIRINOX (leucovorin, 5‐FU, irinotecan, oxaliplatin) is now the most commonly used regimen, with increase in its use driven initially by survival gains seen in metastatic PDAC studies [9, 10]. However, this regimen is associated with increased toxicity, and there is variability in its use in practice [9, 11, 12, 13]. Reflecting these uncertainties, the 2025 National Comprehensive Cancer Network (NCCN) guidelines note a lack of definitive evidence supporting specific neoadjuvant regimens and recommend considering neoadjuvant therapy in the context of a clinical trial [14].

Taken together, we sought to determine if survival differed based on tumor response between patients receiving single‐agent and multi‐agent regimens. We hypothesized that chemosensitivity to neoadjuvant treatment reflects tumor biology and that achieving a pathologic complete response (pCR) with single‐agent therapy may indicate particularly favorable tumor biology.

## 2 Methods

Adult patients with a diagnosis of stage I‐III pancreatic cancer between 2010 and 2021 who received neoadjuvant therapy were identified in the National Cancer Database. Sequencing of neoadjuvant therapy, type of neoadjuvant chemotherapy, and use of radiation were determined using the “RX_SUMM_SYSTEMIC_SUR_SEQ”, “RX_SUMM_CHEMO”, “RX_SUMM_SURGRAD_SEQ” variables, respectively. Treatment regimens were categorized as single‐agent neoadjuvant chemotherapy/chemoradiation (single‐agent), multi‐agent neoadjuvant chemotherapy/chemoradiation (multi‐agent), and multi‐agent neoadjuvant chemotherapy/chemoradiation + adjuvant chemotherapy (multi‐agent+adjuvant). Chemotherapy and chemoradiation groups were combined given comparable OS reported in previous studies [10, 15, 16, 17, 18]. Congruent with these previous reports, our exploratory analysis of the chemotherapy and chemoradiation categories demonstrated similar OS curves (Figure S1). Tumor response was categorized as pathologic complete response (pCR) if the final specimen was pT0N0, moderate response if the final specimen stage decreased by two or greater T‐stage levels from the clinical stage, or minimal/no response if the final specimen stage was the same as the clinical stage or decreased by one T‐stage level from the clinical stage. The minimal and no response categories were combined as their OS curves were nearly superimposable (Figure S2). OS from diagnosis was evaluated for treatment regimens within each tumor response strata. To enable fair comparison with the pCR cohort, patients with positive margins and nodal disease in the final specimen were excluded to minimize the confounder of residual disease burden (Figure S3). A sensitivity analysis including these patients was also performed. Additional variables included age, distance from hospital, and length of stay, which were modeled as continuous variables. For model parsimony, facility type (academic vs. non‐academic), race (White vs. non‐White), insurance status (private vs. non‐private), median income (above vs. below median), tumor location (head vs. body/tail), and Charlson‐Deyo Index (0 vs. ≥ 1) were dichotomized.

### 2.1 Statistical Analyses

Analysis variables were selected *a priori* based on previous literature, clinical knowledge, and availability. Comparisons between variables were performed using the chi‐square test (categorical variables), Student's *t*‐test (continuous variables), or Kruskal‐Wallis test (continuous variables). Unadjusted survival was compared by the Kaplan‐Meier method and log‐rank tests. Adjusted survival analysis was performed using Cox regression. Bidirectional selection considered age, sex, facility type, race, insurance status, income median, tumor location, Charlson‐Deyo Index, clinical stage, hospital length of stay (LOS), distance from a hospital for inclusion in multivariable models. Adjusted hazard ratio controlled for age, sex, race, insurance status, Charlson‐Deyo Index, hospital length of stay, distance from hospital, and clinical stage. Covariate estimates are reported as HRs with 95% confidence intervals (CIs). All tests were two‐sided and assessed for significance at *p* < 0.05 using RStudio (R 4.3.2, Vienna, Austria). This study was deemed exempt from review by the Institutional Review Board.

## 3 Results

### 3.1 Clinicodemographic Characteristics of the Cohort

Of the included cohort, 9.8% achieved complete response, 19.1% had moderate response and 71.1% had minimal/no response. Trends in differences across the groups were intermixed (Table 1). However, compared to patients with minimal/no response, those with complete response were younger (median age 61 [54–69] vs. 64 [57–70] years) and fewer proportion with comorbidities (27% vs. 34%). Median hospital length of stay, 30‐day readmission and 30‐day mortality were similar across response groups.

**Table 1:** Clinicodemographic characteristics of patient cohort by degree of response to neoadjuvant therapy.

	Complete response (*N* = 188)	Moderate response (*N* = 368)	Minimal/No response (*N* = 1367)	*p*
**Age**, median (IQR)	61 (54–69)	63 (57–70)	64 (57–70)	**0.01**
**Sex**, Female (%)	94 (50)	190 (52)	696 (51)	0.93
**Race**, Non‐White (%)	27 (14)	51 (14)	189 (14)	0.99
**Facility Type** (%)				
Academic	121 (65)	226 (62)	856 (63)	0.81
Non‐academic	65 (35)	137 (38)	497 (37)	
**Hospital Distance**, miles, median (IQR)	20 (8–63)	26 (10–71)	23 (23–60)	0.23
**Insurance Status** (%)				
Private	105 (56)	178 (49)	632 (47)	0.05
Non‐Private	82 (43)	185 (51)	720 (53)	
**Income Level**, above median (%)	120 (78)	218 (72)	854 (75)	0.29
**Tumor Location,** Head (%)	133 (77)	1006 (79)	260 (76)	0.49
**Clinical Stage**				**< 0.001**
Stage I	45 (23.9)	1 (0.3)	339 (24.8)	
Stage II	98 (52.1)	218 (59.2)	818 (59.8)	
Stage III	45 (23.9)	149 (40.5)	210 (15.4)	
**Charlson Deyo score** (%)				**0.03**
0	137 (73)	264 (72)	899 (66)	
≥ 1	51 (27)	104 (28)	468 (34)	
**Neoadjuvant Radiation,** *Yes* (%)	118 (63)	251 (68)	752 (55)	**< 0.001**
**Neoadjuvant Approach (%)**				**0.01**
Single‐agent neoadjuvant	21 (11)	37 (10)	190 (14)	
Multi‐agent neoadjuvant	130 (69)	255 (69)	830 (61)	
Multi‐agent neoadjuvant + adjuvant	37 (20)	76 (21)	347 (25)	
**Length of Stay**, Days, median (IQR)	7 (6–10)	7 (6–11)	7 (6–11)	0.99
**30‐day Readmission**, *Yes* (%)	17 (9)	23 (6)	91 (7)	0.43
**30‐day Mortality**, *Yes* (%)	3 (2)	6 (2)	21 (2)	0.99

*Note: p*‐values < 0.05 are bolded.

Treatment patterns also varied modestly by response category. Among patients with complete response, 11% received single‐agent neoadjuvant, 69% received multi‐agent neoadjuvant, and 20% received multi‐agent neoadjuvant plus adjuvant chemotherapy. Similar proportions were observed in the moderate response group (10%, 69%, and 21%, respectively). For those with minimal/no response, 14% received single‐agent neoadjuvant, 61% received multi‐agent neoadjuvant, and 25% received multi‐agent neoadjuvant plus adjuvant chemotherapy. Use of multi‐agent neoadjuvant therapy increased over time (Figure S4).

### 3.2 Overall Survival Outcomes Based on Tumor Response

The 3‐year OS (Figure 1) for the complete response group was 74% (68–81), 56% (51–62) for the moderate response group was, and 50% (47–52) for the minimal/no response group. In multivariable analysis (Table 2), mortality risk was significantly greater for moderate responders (HR 1.70[1.24–2.31], *p* < 0.001) and for minimal/no responders (HR 2.33[1.76–3.08], *p* < 0.001) compared with those achieving complete response. Additional factors independently associated with mortality risk included non‐private insurance, higher clinical stage at diagnosis, and longer hospital length of stay.

**Figure 1:**
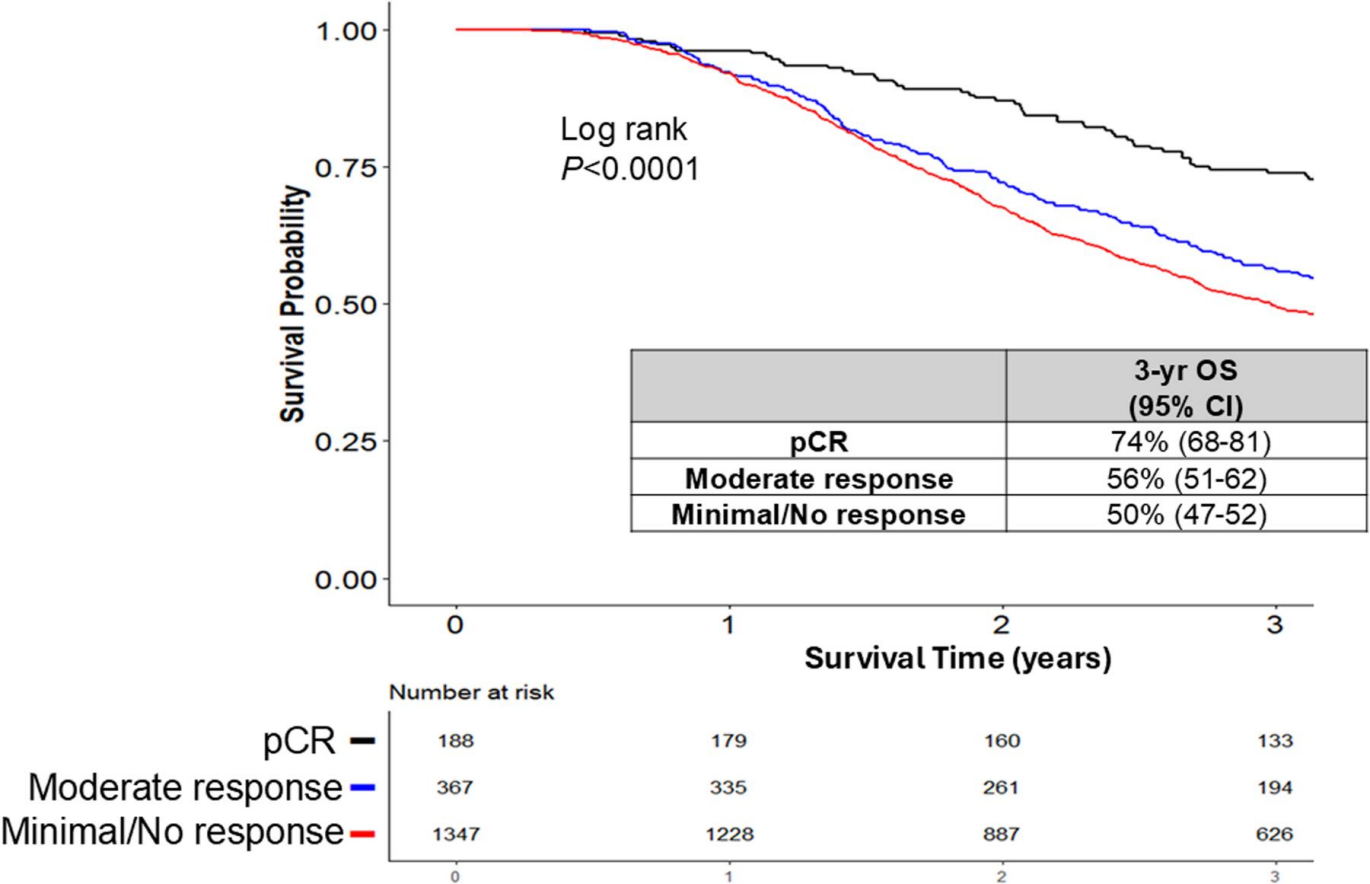
Kaplan‐Meier survival of patients in the cohort based on response to neoadjuvant therapy.

**Table 2:** . Multivariable analysis of factors associated with mortality in the entire cohort.

Variables	Hazard Ratio (95% CI)	*p*
**Age**	1.01 (0.99–1.01)	0.19
**Sex**		
Male	Ref	
Female	1.10 (0.97–1.26)	0.15
**Race**		
White	Ref	
Nonwhite	0.96 (0.79–1.17)	0.70
**Charlson‐Deyo Score**		
0	Ref	
≥ 1	1.06 (0.92–1.22)	0.46
**Insurance**		
Non‐private	Ref	
Private	0.81 (0.69–0.95)	**0.01**
**Hospital Distance**	1.00 (1.00–1.00)	0.06
**Length Of Stay**	1.01 (1.00–1.02)	**< 0.01**
**Stage**		
I	Ref	
II	1.62 (1.33–1.98)	**< 0.001**
III	2.25 (1.79–2.84)	**< 0.001**
**Neoadjuvant Response**		
Complete	Ref	
Moderate	1.70 (1.24–2.31)	**0.001**
Minimal/None	2.33 (1.76–3.08)	**< 0.001**

*Note: p*‐values < 0.05 are bolded.

### 3.3 Overall Survival Outcomes Based on Chemotherapy Regimen Received

We next stratified each response category by neoadjuvant regimen received (Tables S1–S3). Among patients who achieved complete response (Figures 2), 3‐yr OS was 81% [66–99] for single‐agent neoadjuvant, 69% [61–78] for multi‐agent neoadjuvant, and 87% [76–98] for multi‐agent neoadjuvant plus adjuvant therapy. On multivariable Cox analysis, survival did not significantly differ among the three treatment groups. Furthermore, exploratory analysis comparing those who received multi‐agent neoadjuvant chemotherapy to those who received multi‐agent neoadjuvant chemoradiation within the pCR cohort revealed similar OS (Figure S5). In contrast, neoadjuvant regimen was strongly associated with survival among moderate (Figure 3) and minimal/no response groups (Figure 4). For the moderate responders, receipt of single‐agent neoadjuvant was associated with significantly higher mortality risk (HR 2.05 [1.16–3.63]) compared to multi‐agent neoadjuvant plus adjuvant therapy after adjusting for confounders. Similarly, in the minimal/no response group, single‐agent therapy was associated with higher mortality risk (HR 1.54 [1.20–1.97]) compared with multi‐agent neoadjuvant plus adjuvant therapy, after adjusting for confounders. In sensitivity analysis, OS trend remained stable upon inclusion of patients with nodal metastasis and positive margins (Figure S6).

**Figure 2:**
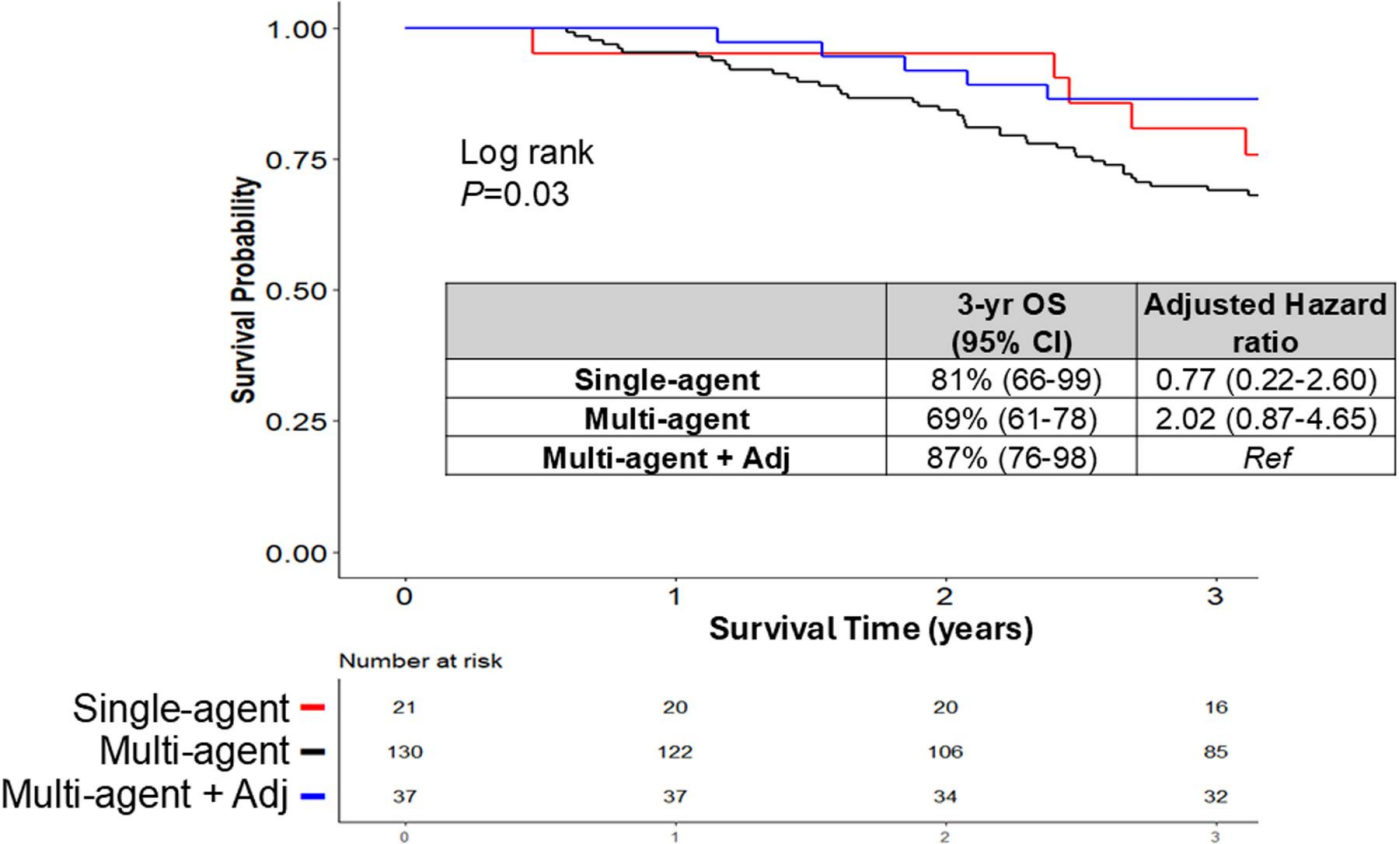
Kaplan‐Meier survival among various neoadjuvant regimen among patients with pathologic complete response. Inset shows 3‐yr overall survival and hazard ratio after adjusting for confounders.

**Figure 3:**
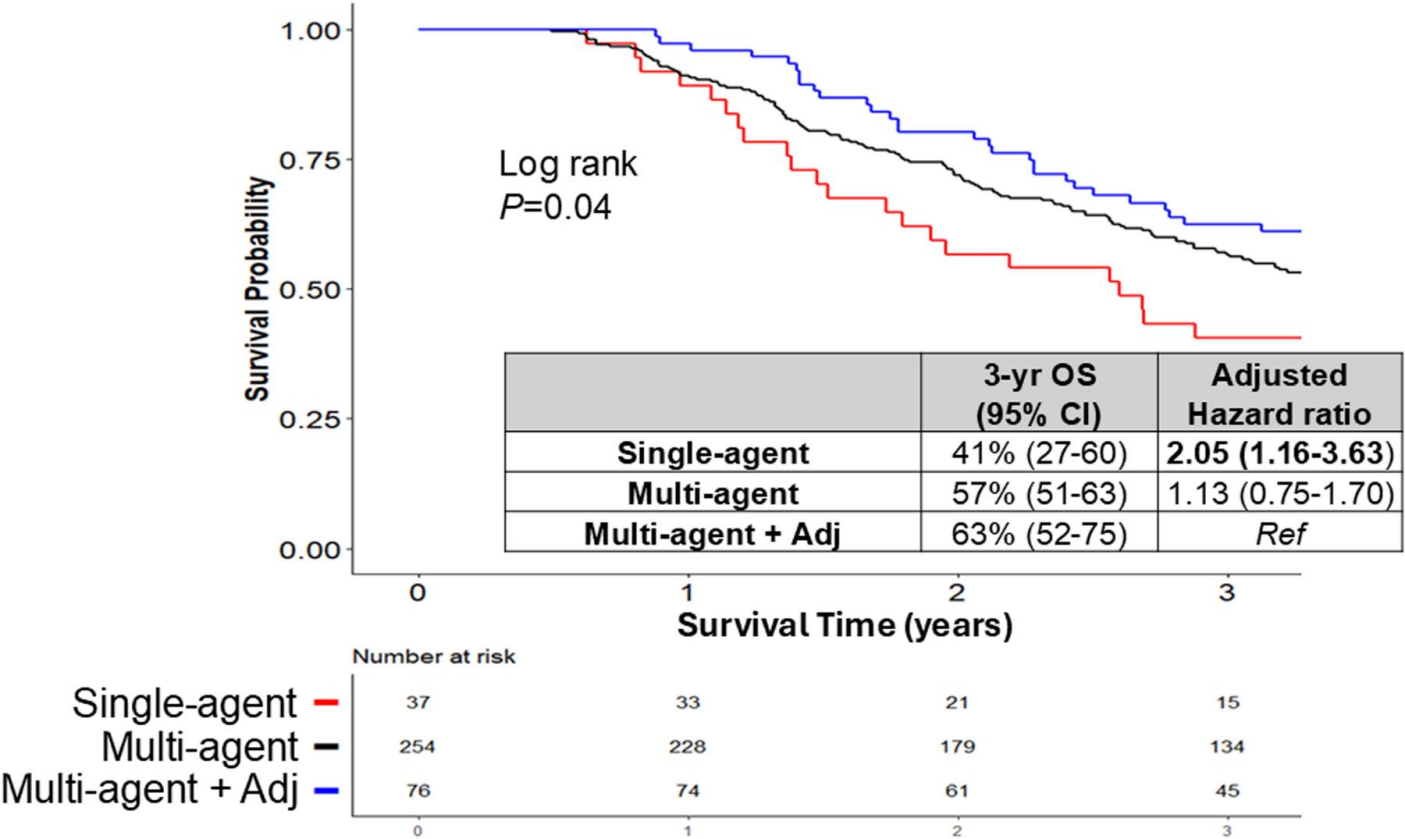
Kaplan‐Meier survival among various neoadjuvant regimen among patients with moderate response. Inset shows 3‐yr overall survival and hazard ratio after adjusting for confounders.

**Figure 4:**
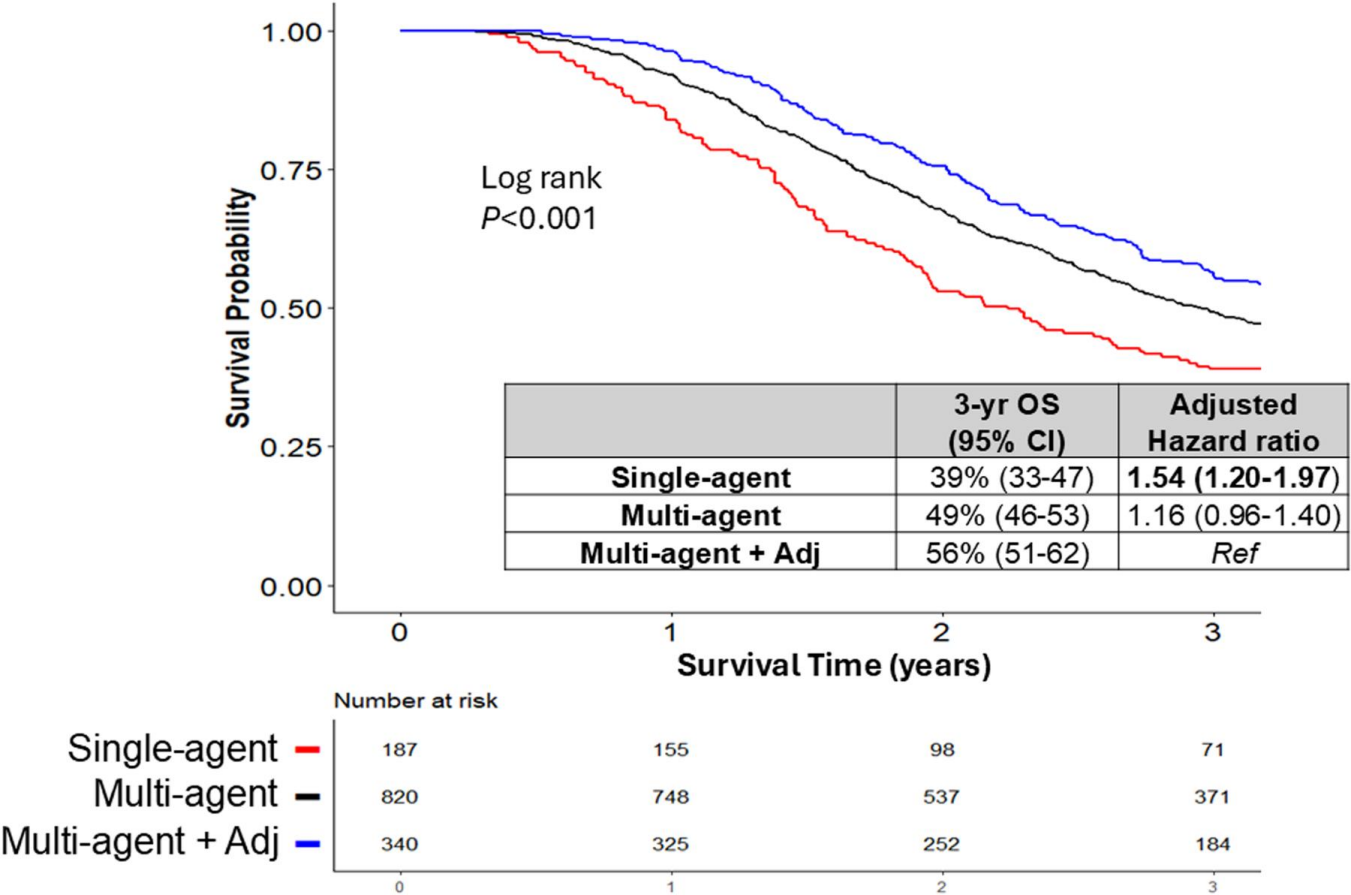
Kaplan‐Meier survival among various neoadjuvant regimen among patients with minimal/no response. Inset shows 3‐yr overall survival and hazard ratio after adjusting for confounders.

## 4 Discussions

In this study, we found that patients with pCR had significantly improved OS compared to those with moderate or minimal/no response to neoadjuvant therapy. Among patients with moderate or minimal/no response, single‐agent neoadjuvant was associated with increased mortality risk. On the other hand, a small subset (~11%) of patients achieved pCR with single‐agent neoadjuvant therapy and had survival comparable to those who received multi‐agent plus adjuvant therapy, even after adjusting for confounders. These findings highlight the biological heterogeneity of PDAC and suggest that neoadjuvant therapy response may serve as a window to distinct molecular subtypes of good versus bad tumor biology. Furthermore, two key practical considerations emerge. First, given the low pCR rates for PDAC, further investigation into optimal neoadjuvant regimens remains essential. Enrollment in clinical trials should be encouraged as recommended by NCCN guidelines [14]. Secondly, as emerging evidence suggest, improving PDAC outcomes will likely depend on improved molecular characterization [19]. Molecular profiling by response type and type of neoadjuvant regimen utilized may help identify predictive biomarkers to guide personalized neoadjuvant selection.

Despite the benefits of neoadjuvant approach purported across several studies, the regimen of choice remains debated. The recent PREOPANC‐2 trial compared multi‐agent neoadjuvant chemotherapy (FOLFIRINOX) to neoadjuvant single‐agent chemoradiation (gemcitabine with hypofractionated radiotherapy) plus adjuvant chemotherapy [10]. OS and PFS were similar for both groups. Although the pCR rate was numerically higher in the multi‐agent arm, the difference was not statistically significant (10% vs. 5%, *p* = 0.25). Although variable, it should be noted that the contemporary estimated pCR rate for PDAC following neoadjuvant therapy is approximately 3%–4% [20]. The higher pCR rate observed in our study (9.8%) is likely largely attributable to cohort selection, as patients with node positive and margin positive resection were excluded to facilitate comparison with the pCR cohort. When these patients are included, the pCR rate decreases to ~4.9% which is comparable to contemporary rates. One recent study published in JAMA that evaluated patients with node negative PDAC similarly found higher pCR rates than previously reported at 6.4% [21]. Notably, our objective was not to estimate population incidence of pCR but rather to evaluate survival outcomes among patients exhibiting varying degrees of pathologic response.

Previous studies have also examined outcomes among patients achieving pCR. He et al. (2018) performed a single institution assessment of patients with PDAC who achieved pCR [22]. The pCR cohort consisted of 19 patients, and 3 (~16%) achieved pCR on a single‐agent regimen. They categorized other response categories in their study as “near complete” and “limited”. “Near complete” response was defined as less than 1 cm of tumor without nodal metastasis while all other patients were deemed limited responders. They found OS and DFS to be significantly better in the pCR group compared to the other two groups. Sell et al. (2020) utilized the NCDB data from 2004 to 2014 and reported similar findings [23]. Their pCR cohort comprised of 41 patients. Of note, our study expands on this literature in several important ways. Briefly, we utilized a more contemporary dataset with a larger cohort. Additionally, we specifically evaluated survival differences across neoadjuvant regimen types within each response category. Finally, our response classification of moderate and minimal/no response groups had clearer survival curve discrimination (compared to the previously reported near‐complete and limited/incomplete response definitions) and likely better reflects true heterogeneity in PDAC tumor biology.

The role of adjuvant therapy in addition to multi‐agent therapy remains unclear. In a multicenter international retrospective cohort of 520 patients treated with neoadjuvant FOLFIRINOX, van Roessel et al. (2020) found survival benefit from adjuvant chemotherapy only among node‐positive patients [24]. Conversely, Olecki et al. (2021), using NCDB data from 2006 to 2015 found that additional adjuvant therapy was associated with improved OS only in those with favorable pathologic features such as negative nodes, low lymph node ratio, low‐grade histology, and negative surgical margins [25]. Differences in regimen heterogeneity and patient selection may explain these contrasting findings. For example, all patients in the study by van Roessel et al. received FOLFIRINOX while the regimen in the study by Olecki was more diverse. In our study, patients with nodal involvement were excluded and OS was similar for the neoadjuvant multi‐agent only and neoadjuvant multi‐agent plus adjuvant groups across all response categories.

Our decision to combine chemotherapy‐only and chemoradiation cohorts warrants further discussion. Firstly, we would like to point out that our primary outcome of interest is OS, which a preponderance of retrospective and prospective studies suggest is not significantly different with the addition of radiation [10, 15, 16, 17, 18, 26]. More recently, the CONKO‐007 trial demonstrated no OS advantage with the addition of neoadjuvant chemoradiation following multi‐agent chemotherapy [27]. Consistent with these observations, stratified analyses within our cohort demonstrated near identical survival curves between chemotherapy‐only and chemoradiation‐treated patients. Of note, the single‐agent group is unique because single‐agent chemotherapy alone is unlikely to be used in the current era. Unsurprisingly, over 80% of the single‐agent group received chemoradiation. Given the absence of meaningful survival difference between the chemotherapy and chemoradiation groups and our focus of overall survival, we elected to combine these cohorts to improve statistical power, especially as the single‐agent chemotherapy patients still had the requisite tumor response to be included.

Several limitations should be acknowledged. Briefly, our study is subject to risk of selection bias and data missingness inherent to retrospective studies. Exclusion of patients with nodal metastasis and positive margins was performed to facilitate a more biologically comparable assessment of treatment response but may limit generalizability of our findings. Nevertheless, sensitivity analyses including these patients demonstrated that overall trends remained consistent. However, it is also worth noting that the proportion of patients achieving node and margin negative disease has increased in contemporary practice due to neoadjuvant therapy making this cohort clinically relevant for evaluating the prognostic implications of treatment response [21, 28, 29, 30]. Although T‐stage downstaging is convenient for assessing response particularly as neoadjuvant use is increasingly associated with higher rates of node negative disease, T‐stage downstaging alone may not fully capture biologic response especially as treatment‐associated fibrosis and radiation effects can alter pathologic staging independent of viable tumor burden [31, 32, 33, 34, 35, 36]. This limitation may partly explain the paradox as to why neoadjuvant chemoradiation is associated with increased pCR rates but not OS despite pCR being associated with better OS. Also, the minimal and no response groups were combined because of overlapping survival curves, which was our primary outcome of interest. However, it is plausible that these combined groups differ in other clinically meaningful outcomes not explored in our study. Finally, key granular details such as specific chemotherapy regimens, treatment duration, and cancer‐specific survival are not collected by the NCDB. Despite these limitations, the NCDB remains a robust and well‐validated resource with data coverage of nearly 70% of newly diagnosed cancer cases in the US.

In summary, our findings suggest that tumor chemosensitivity during neoadjuvant therapy reflects tumor biology, and achieving pCR with single‐agent neoadjuvant may indicate favorable tumor biology. Nevertheless, most patients do not achieve pCR and multi‐agent neoadjuvant therapy is associated with improved survival in those patients. Future biomarker‐driven translational studies are needed to identify molecular signatures of favorable biology and guide personalized neoadjuvant de‐escalation strategies in PDAC.

## 5 Synopsis

In this national database study, response to neoadjuvant therapy was associated with overall survival, with patients who achieve pCR having the best outcomes. Most patients do not achieve pCR and multi‐agent neoadjuvant therapy is associated with improved prognosis in these patients.

## Supporting information


Supporting File

## Data Availability

The data that support the findings of this study are available in National Cancer Database at https://www.facs.org/quality-programs/cancer-programs/national-cancer-database/puf/. These data were derived from the following resources available in the public domain‐NCDB, https://www.facs.org/quality-programs/cancer-programs/national-cancer-database/ncdb-data-submission. The data used in the study are derived from a de‐identified National Cancer Database. The American College of Surgeons and the Commission on Cancer have not verified and are not responsible for the analytic or statistical methodology employed, or the conclusions drawn from these data by the investigator. All data that support this study's findings are available from the corresponding author upon reasonable request.
