# Supplementary material for: Impact of Neoadjuvant Approach and Tumor Response on Survival in Pancreatic Adenocarcinoma
Source: J Surg Oncol. 2026 Sep 3;134(5):631–8. doi: 10.1002/jso.70329 (PMC13619096; doi:10.1002/jso.70329)
Supplement: Supplementary file 1 [file JSO-134-631-s001.docx]

**Supplemental Figure 1:** Overall survival curves are similar for single-agent chemotherapy vs. single-agent chemoradiation (A), multi-agent chemotherapy vs. multi-agent chemoradiation (B), multi-agent chemotherapy + Adjuvant vs. multi-agent chemoradiation + Adjuvant (C). Analysis inclusive of patients with nodal metastasis and positive margins.

**Supplemental Figure 2:** Overall survival curves are similar for minimal vs. no response categories following neoadjuvant therapy in the entire cohort. Analysis inclusive of patients with nodal metastasis and positive margins.

**Supplemental Figure 3:** CONSORT diagram of patient selection criteria in this study.

**Supplemental Figure 4:** Trend in the use of multiagent vs. single agent neoadjuvant in the selected cohort.

**Supplemental Figure 5:** Kaplan-Meier survival among pCR patients who received neoadjuvant multi-agent chemotherapy vs. multi-agent neoadjuvant chemoradiation.

**Supplemental Figure 6:** Kaplan-Meier survival among moderate (A) and minimal/no responders (B) inclusive of patients with nodal metastasis and positive margins.

**Supplemental Table 1:** Clinicodemographic characteristics by regimen among patients with complete response.

|  | Single-agent neoadjuvant (N=21) | Multi-agent neoadjuvant (N=130) | Multi-agent neoadjuvant + adjuvant chemo  (N=37) | *P-*value |
| --- | --- | --- | --- | --- |
| **AGE**, median (IQR) | 60 (52-69) | 62 (55-70) | 59 (53-65) | 0.19 |
| **SEX**, Female (%) | 13 (62) | 62 (48) | 19 (51) | 0.47 |
| **RACE**, Non-White (%) | 8 (38) | 18 (14) | 1 (3) | **0**.**001** |
| **FACILITY TYPE** (%) | 9 (43) | 44 (34) | 12 (32) |  |
| Academic | 12 (57) | 84 (66) | 25 (68) | 0.71 |
| Non-academic | 9 (43) | 44 (34) | 12 (32) |  |
| **HOSPITAL DISTANCE**, miles, median (IQR) | 20 (7-90) | 18 (8-61) | 38 (13-73) | 0.64 |
| **INSURANCE STATUS** (%) |  |  |  |  |
| Private | 10 (50) | 68 (52) | 27 (73) | 0.07 |
| Non-Private | 10 (50) | 62 (48) | 10 (27) |  |
| **INCOME LEVEL**, above median (%) | 6 (33.3) | 23 (21.7) | 5 (16.7) | 0.40 |
| **TUMOR LOCATION,** Head (%) | 17 (90) | 89 (74) | 27 (79) | 0.31 |
| **CLINICAL STAGE** |  |  |  | 0.95 |
| Stage I | 5 (24) | 30 (23) | 10 (27) |  |
| Stage II | 11 (52) | 67 (52) | 20 (54) |  |
| Stage III | 5 (24) | 33 (25) | 7 (19) |  |
| **CHARLSON DEYO SCORE** (%) |  |  |  | 0.46 |
| 0 | 15 (71) | 92 (71) | 30 (81) |  |
| ≥1 | 6 (29) | 38 (29) | 7 (19) |  |
| **NEOADJUVANT RADATION,** *Yes* (%) | 17 (81) | 84 (65) | 17 (46) | **0.02** |
| **LENGTH OF STAY**, Days, median (IQR) | 8 (7-9) | 8 (6-11) | 7 (6-8) | 0.16 |
| **30-DAY READMISSION**, *Yes* (%) | 0 (0) | 14 (11) | 3 (8) | 0.28 |
| **30-DAY MORTALITY**, *Yes* (%) | 0 (0) | 3 (2) | 0 (0) | 0.50 |

**Supplemental Table 2:** Clinicodemographic characteristics by regimen among patients with moderate response.

|  | Single-agent neoadjuvant (N=37) | Multi-agent neoadjuvant (N=255) | Multi-agent neoadjuvant + adjuvant chemo  (N=76) | *P-*value |
| --- | --- | --- | --- | --- |
| **AGE**, median (IQR) | 63 (59-68) | 64 (58-70) | 61 (55-79) | 0.10 |
| **SEX**, Female (%) | 17 (46) | 136 (53) | 37 (49) | 0.60 |
| **RACE**, Non-White (%) | 6 (16) | 38 (15) | 7 (9) | 0.42 |
| **FACILITY TYPE** (%) |  |  |  |  |
| Academic | 23 (62) | 162 (64) | 41 (55) | 0.38 |
| Non-academic | 14 (38) | 90 (36) | 33 (45) |  |
| **HOSPITAL DISTANCE**, miles, median (IQR) | 45 (7-169) | 28 (10-70) | 20 (9-50) | 0.28 |
| **INSURANCE STATUS** (%) |  |  |  |  |
| Private | 18 (49) | 119 (48) | 41 (54) | 0.62 |
| Non-Private | 19 (51) | 131 (52) | 35 (46) |  |
| **INCOME LEVEL**, above median (%) | 7 (25) | 63 (29) | 16 (26) | 0.83 |
| **TUMOR LOCATION,** Head (%) | 29 (85) | 185 (75) | 50 (76) | 0.42 |
| **CLINICAL STAGE** |  |  |  | 0.37 |
| Stage II | 19 (51) | 147 (58) | 52 (68) |  |
| Stage III | 18 (49) | 107 (42) | 24 (32) |  |
| **CHARLSON DEYO SCORE** (%) |  |  |  | 0.59 |
| 0 | 24 (65) | 186 (73) | 54 (71) |  |
| ≥1 | 13 (35) | 69 (27) | 22 (29) |  |
| **NEOADJUVANT RADATION,** *Yes* (%) | 34 (92) | 180 (71) | 37 (49) | **<0.001** |
| **LENGTH OF STAY**, Days, median (IQR) | 8 (5-9) | 8 (6-12) | 7 (6-8) | **0.01** |
| **30-DAY READMISSION**, *Yes* (%) | 1 (3) | 18 (7) | 4 (5) | 0.54 |
| **30-DAY MORTALITY**, *Yes* (%) | 0 (0) | 6 (2) | 0 (0) | 0.26 |

**Supplemental Table 3**: Clinicodemographic characteristics by regimen among patients with minimal/no response

|  | Single-agent neoadjuvant (N=190) | Multi-agent neoadjuvant (N=830) | Multi-agent neoadjuvant + adjuvant chemo  (N=347) | *P-*value |
| --- | --- | --- | --- | --- |
| **AGE**, median (IQR) | 65 (57-72) | 64 (58-71) | 63 (56-69) | **0.001** |
| **SEX**, Female (%) | 100 (53) | 423 (51) | 173 (50) | 0.83 |
| **RACE**, Non-White (%) | 30 (16) | 111 (14) | 48 (14) | 0.72 |
| **FACILITY TYPE** (%) |  |  |  |  |
| Academic | 121 (64) | 516 (63) | 219 (64) | 0.92 |
| Non-academic | 68 (36) | 305 (37) | 124 (36) |  |
| **HOSPITAL DISTANCE**, miles, median (IQR) | 18 (9-50) | 25 (9-65) | 20 (9-53) | 0.29 |
| **INSURANCE STATUS** (%) |  |  |  |  |
| Private | 75 (40) | 368 (45) | 189 (55) | **0.001** |
| Non-Private | 111 (60) | 453 (55) | 156 (45) |  |
| **INCOME LEVEL**, above median (%) | 48 (29) | 168 (24) | 65 (23) | 0.30 |
| **TUMOR LOCATION,** Head (%) | 151 (84) | 604 (78) | 251 (78) | 0.17 |
| **CLINICAL STAGE** |  |  |  | 0.11 |
| Stage I | 51 (27) | 206 (25) | 82 (24) |  |
| Stage II | 111 (58) | 482 (58) | 225 (65) |  |
| Stage III | 28 (15) | 142 (17) | 40 (11) |  |
| **CHARLSON DEYO SCORE** (%) |  |  |  | 0.44 |
| 0 | 118 (62) | 555 (67) | 226 (65) |  |
| ≥1 | 72 (38) | 275 (33) | 121 (35) |  |
| **NEOADJUVANT RADATION,** *Yes* (%) | 157 (83) | 438 (53) | 157 (45) | **<0.001** |
| **LENGTH OF STAY**, Days, median (IQR) | 8 (6-12) | 8 (6-11) | 7 (6-9) | **<0.001** |
| **30-DAY READMISSION**, *Yes* (%) | 15 (8) | 57 (7) | 19 (6) | 0.52 |
| **30-DAY MORTALITY**, *Yes* (%) | 5 (3) | 15 (2) | 1 (0.3) | 0.07 |
